# Supplementary material for: Proposal of Patescibacterium danicum gen. nov., sp. nov. in the ubiquitous bacterial phylum Patescibacteriota phyl. nov
Source: ISME Commun. 2024 Nov 19;5(1):ycae147. doi: 10.1093/ismeco/ycae147 (PMC11809585; doi:10.1093/ismeco/ycae147)
Supplement: Supplementary_Text_ycae147 [file supplementary_text_ycae147.pdf]

Supplementary Text of the manuscript

"Proposal of *Patescibacterium danicum* gen. nov., sp. nov. in the ubiquitous bacterial phylum *Patescibacteriota* phyl. nov. "

Content:

1) ***MIMAG standards***
2) ***The importance of defining type material***
3) ***SingleM detection of Patescibacteriota with reduced genome sizes***
4) ***Gene annotation of Patescibacteriota***
5) ***16S rRNA sequence of ABY1 and Fred.cMAG.1***

(1) MIMAG standards

We evaluated the 10 MAGs in our study according to MIMAG standards. Thereby the MIMAG standards for high-quality draft MAGs are defined as follows. Assembly quality: multiple fragments where gaps span repetitive regions. Presence of the 23S, 16S, and 5S rRNA genes and at least 18 tRNAs. Estimated completion >90%; Estimated contamination <5%. For details see (Bowers et al. 2017).

Table ST1 | Evaluation of the 10 MAGs according to MIMAG standards for high-quality draft MAGs. MAGs that qualify are highlighted in bold in the second column. Quality metrics that disqualify MAGs are highlighted in red.

Data source	MAG / acc. no.	tRNAs	Genome (bp)	Con (#)	Comp (%)	Con (%)	GC (%)	CD (%)	Prot (#)	5-16-23 (#)
Singleton et al. 2021	**ABY1^{TS} (GCA_016699775.1)**	18	938,851	1	96.03*	0.69	35.4	88.2	890	1-1-1
This study	Fred.cMAG.1 (GCA_964214775.1)	17	917,454	1	95.89*	0.9	35.4	85	978	1-1-1
NCBI	**GCA_002344425.1**	18	752,947	38	95.69	0.53	43.9	92.3	792	1-1-1
NCBI	GCA_002433955.1	17	748,244	38	91.4	0.93	43.9	91.2	777	1-2-1
NCBI	GCA_002293885.1	15	690,185	59	*89.88*	0.71	44.2	90.4	728	1-0-1
NCBI	GCA_002343995.1	16	746,423	24	*95.51*	1.79	43.9	93.1	774	2-1-1
IMG/M	IMGM3300014059_BIN201	15	912,772	53	*90.14*	0.32	36.1	89.5	959	0-1-1
IMG/M	**IMGM3300014204_BIN854**	19	962,299	12	*96.48*	0.53	35.2	90.8	973	1-1-1
IMG/M	**IMGM3300029288_BIN286**	18	915,973	10	*96.74*	0.85	35.2	88.7	916	1-1-1
IMG/M	IMGM3300030493_BIN257	13	530,683	60	*69.2*	0.23	34.9	92.2	583	0-1-0

Reference

Bowers, Robert M., Nikos C. Kyrpides, Ramunas Stepanauskas, Miranda Harmon-Smith, Devin Doud, T. B. K. Reddy, Frederik Schulz, et al. 2017. 'Minimum Information about a Single Amplified Genome (MISAG) and a Metagenome-Assembled Genome (MIMAG) of Bacteria and Archaea'. Nature Biotechnology 35 (8): 725–31. https://doi.org/10.1038/nbt.3893.

(2) The importance of defining type material

Defining type material is a fundamental principle in naming biological taxa, and is standard practice in botanical, zoological, and prokaryotic nomenclature. Indeed, the nomenclature of prokaryotes, and the associated principle of declaring type material, has been regulated by the rules of the International Code of Nomenclature of Prokaryotes (ICNP) over the last thirty years (Lapage et al. 1992). The ICNP requires, as of to date, that a strain of a cultivable bacterial or archaeal species has to be deposited in a permanent culture collection to qualify as nomenclatural type (Oren 2023). Over the last two decades, advances in metagenomics and single-cell genomics have created an overwhelming number of prokaryotic genomes that have allowed discoveries and descriptions of many uncultured lineages. However, these genomes were deemed not acceptable to serve as type material for the valid publication of names under the ICNP. Recently, this *status quo* has been challenged by a proposal to allow sequencing data to serve as type material (Whitman 2016; Whitman, Sutcliffe, and Rossello-Mora 2019), which was formally rejected by the long-standing International Committee on Systematics of Prokaryotes (ICSP) (Oren 2023). The result was a community driven initiative that eventually developed the SeqCode, a code of nomenclature under which genome sequences serve as nomenclatural types (Hedlund et al. 2022). This code enables valid publication of names of prokaryotes based on metagenome-assembled genome, single-amplified genome sequences, or isolate genomes as type material.

With this paper we contributed a modest step towards nomenclatural and taxonomic stability of the phylum Patescibacteriota by designate the type genome ABY1^{TS} and by proposing the species *Patescibacterium* danicum sp. nov., the genus *Patescibacterium* gen. nov, and associated higher ranks, including the phylum *Patescibacteriota* phyl. nov., according to the SeqCode. In doing so, we used the public SeqCode Registry (https://seqco.de), a registration portal that guides the user through the type genome and naming process, followed by a genome quality and nomenclature review to guarantee that the submission follows the SeqCode rules.

Note that we use the superscript "Ts" that stands for the type genome of the species according to the SeqCode recommendations (see Chapter 4; https://registry.seqco.de/page/seqcode). This is proposed as a way to distinguish the nature of the types, e.g. ICNP uses superscript "T" following the species name (if the sequence of type strain was used in the analyses) or the strain identifier. In table 1, we used "TS" = designated type genome of the species, in analogy with the type strains under the ICNP.

Does a type, e.g. the type species *P. danicum* TS, need to have typical characteristics of associated higher ranks?

According to prokaryotic nomenclature rules, the type does not need to be the most typical member or a representative of the taxon (e.g., type genus of the phylum), but it should be unambiguous (Rule 15, ICNP; Oren et al., IJSEM 2023; Principle 5, SeqCode; https://registry.seqco.de/page/seqcode). In other words, the type only needs to be distinct to function as an indispensable point of reference.

However, based on our genomic characterization of the genus *Patescibacterium* and its type species *P. danicum* TS, we concluded that this type genus is representative of the phylum. It possesses typical characteristics of other members such as reduced genomes size, distinct inferred functions and a predicted host dependent lifestyle. This conclusion is supported by the presence-absence pattern of predicted functions that revealed correlations of *P. danicum* and the representative of its class and most other classes in the phylum (**Fig. S15**).

References

Hedlund, Brian P., Maria Chuvochina, Philip Hugenholtz, Konstantinos T. Konstantinidis, Alison E. Murray, Marike Palmer, Donovan H. Parks, et al. 2022. 'SeqCode: A Nomenclatural Code for Prokaryotes Described from Sequence Data'. *Nature Microbiology* 7 (10): 1702–8. https://doi.org/10.1038/s41564-022-01214-9.

Lapage, S. P., P. H. A. Sneath, E. F. Lessel, V. B. D. Skerman, H. P. R. Seeliger, and W. A. Clark, eds. 1992. *International Code of Nomenclature of Bacteria: Bacteriological Code, 1990 Revision*. Washington (DC): ASM Press. http://www.ncbi.nlm.nih.gov/books/NBK8817/.

Oren, Aharon. 2023. 'Naming New Taxa of Prokaryotes in the 21st Century'. *Canadian Journal of Microbiology* 69 (4): 151–57. https://doi.org/10.1139/cjm-2022-0266.

Oren, Aharon, David R. Arahal, Markus Göker, Edward R. B. Moore, Ramon Rossello-Mora, and Iain C. Sutcliffe. 2023. 'International Code of Nomenclature of Prokaryotes. Prokaryotic Code (2022 Revision)'. *International Journal of Systematic and Evolutionary Microbiology* 73 (5a): 005585. https://doi.org/10.1099/ijsem.0.005585.

Whitman, William B. 2016. 'Modest Proposals to Expand the Type Material for Naming of Prokaryotes'. *International Journal of Systematic and Evolutionary Microbiology* 66 (5): 2108–12. https://doi.org/10.1099/ijsem.0.000980.

Whitman, William B., Iain C. Sutcliffe, and Ramon Rossello-Mora. 2019. 'Proposal for Changes in the International Code of Nomenclature of Prokaryotes: Granting Priority to Candidatus Names'. *International Journal of Systematic and Evolutionary Microbiology* 69 (7): 2174–75. https://doi.org/10.1099/ijsem.0.003419.

3) ***SingleM detection of Patescibacteriota with reduced genome sizes***

The tool singleM (CIT) has the ability to detect small genomes, including symbionts with highly reduced genomes (<300kb). These tiny genomes, i.e. having about ⅓ of the genome size of Patescibacteriota, contained fewer marker genes than average (> 54% of the bacterial markes) but remained detectable despite their extensive gene loss, although their abundances were underestimated by the current implementation of SingleM.

To assess if *Patescibacteriota* genomes contained considerably fewer marker genes, we compared the number of singleM markers present in the *Patescibacterium danicum* genomes and genomes of other *Patescibacteriota* classes, against the maximum of 35 bacterial markers used by singleM. The results show that *Patescibacteriota* class representatives contain on average 92.1% (32.2 ± 3.01) of the singleM marker genes (**Table ST2**), which is well above the 10% threshold implemented by singleM:
"The abundance of each taxon is calculated as a trimmed mean taken across the marker genes, excluding those with total abundance in the lowest and highest 10% to account for taxonomy misassignment and lineages with reduced genomes that do not encode all marker genes."

Table ST2 | SingleM markers detected across Patescibacteriota genomes. Shown are the number of bacterial marker genes (max. 35) detected in each genome representative of the 24 *Patescibacteriota* classes present in GTDB r214 and the genomes of the proposed type species *P. danicum* ABY1TS (GCA_016699775.1) and Fred.cMAG.1 (GCA_964214775.1). The estimated completeness by CheckM2 is also provided for each genome. The number of bacterial singleM markers was calculated with the command [1) singlem pipe --genome-fasta-files file.fna --otu-table output.file 2) singlem summarise --exclude-off-target-hits --input-otu-tables otu_input --output-otu-table otu_output]

**Genome**	**Number of bacterial marker genes out of 35**	**Completeness (CheckM2)**
ABY1TS (GCA_016699775.1) *P. danicum* genome	30	96.03%
Fred.cMAG.1 (GCA_964214775.1) Patescibacteriota genome	31	95.89%
c__Paceibacteria (GCA_000995965)	34	97.03%
c__Kazan-3B-28 (GCA_001029795)	32	97.05%
c__Gracilibacteria (GCA_001430755)	35	98.14%
c__CPR3 (GCA_001771135)	35	97.18%
c__Andersenbacteria (GCA_001817055)	33	95.63%
c__ABY1 (GCA_001818315)	34	100.0%
c__GCA-2792135 (GCA_002792135)	30	85.23%
c__CPR2_A (GCA_002792735)	35	97.01%
c__Dojkabacteria (GCA_002840365)	33	98.54%
c__Saccharimonadia (GCA_004138405)	33	69.8%
c__SOKK01 (GCA_004374725)	30	77.95%
c__SICC01 (GCA_009694835)	23	82.81%
c__JACMRA01 (GCA_014377045)	31	85.86%
c__JAHJAL01 (GCA_018814055)	24	81.69%

c__JABMPQ01 (GCA_018818205)	35	97.09%
c__4484-211 (GCA_018824115)	33	95.76%
c__DYJS01 (GCA_020723005)	32	91.46%
c__UBA1384 (GCA_021157605)	32	96.04%
c__JACPGU01 (GCA_022703645)	33	99.28%
c__CPR2 (GCA_023473595)	31	84.64%
c__Doudnabacteria (GCA_023484515)	33	97.77%
c__Microgenomatia (GCA_024277325)	33	94.38%
c__CG2-30-54-11 (GCA_903889905)	35	97.38%
c__WWE3 (GCA_943354835)	34	100.0%
c__JAEDAM01 (GCF_021057185)	33	100.0%
**Average and Standard deviation (all)**	**32.1**	**2.94**
**Average and Standard deviation (class only)**	**32.24**	**3.01**

4) Gene annotation of Patescibacteriota genomes

We have compared Prokka, Bakta and BlastKOALA for the annotation of *Patescibacteriota* genomes (*see Methods*). All three tools provided comparable results, however Bakta and to some degree also Prokka were missing important KEGG annotations that were recovered with BlastKOALA.

We made the Bakta developers aware of this fact, and they replied that additional information, like dbxrefs, ECs, etc., will make it into the Bakta annotation database soon, thus improving the annotation outcome.

We also used gapseq to find additional encoded transporters that were not recovered by the other tools.

5) 16S rRNA sequence of ABY1 and Fred.cMAG.1

Note that, the 16S rRNA genes of the two MAGs (ABY1^{TS} and Fred.cMAG.1) assigned to *P. danicum* were identical, hence there is only one sequence provided:

>ABY1__16S_rRNA_gene_1486bp
ATCACAGAGAGTTTGATCCTGGCTCAGGATGAACGCTTGCGATGTGTCTAAGGCATGCAAGTCGAATGCTAGTAG
CAATACTGGCATGGCAAACGGGAGAGTAACACATTGGTAACTTACCTCGAAGTCGGGCATAACCCGGAGAAATC
CGGAGTAATTCCCGATGGCCGTGGGGGGACATAAGTCATTCCCACGTAAAGTTTTTTCGCTTCGAGAGAGACCT
ATGTTCTATCAGCTAGTTGGTAAGGTAACGGCTTACCAAGGCTACGACGGATAGCGGGTGTGAGAGCATGGCCC
GCCTCACTGGGACTGAGAAACTGCCCAGACTCCTACGGGAGGCTGCAGTCAAGAATCTTCCTCAATGGCCGAAA

GGCTGAAGGAGCGACATCGCGTGCAGGAGGAAGCCCTTCGGGGTGTAAACTGCTTTTCTCTGGGAAGAAACAAT
GACGGTACCAGAGGAATAAGGGGTTGCTAAACTCGTGCCAGCAGCAGCGGTAATACGAGTACCCCGAGCGTTAT
CCGGATTTATTGGGCGTAAAGCGTGCGTAGACGGTTTATAACATCTCCTGTTAAATCCCAAGGCTCAACCTTGGG
GCTGCGGGAGATATGGATAAACTAGAGGCTGGAAGAGGTAAGCGGAATTGCTGGTGTAGGGGTTAAATCCGTTA
ATATCAGCAGGAACACCAAATGCGAAGGCAGCTTACTGGAACAGTTCTGACGTTCAGGCACGAAAGCGTGGGGA
GCGAATGGGATTAGATACCCCAGTAGTCCACGCTGTAAACGATGGATGCTAAGCATTGGGAGTTTCGACCCTCT
CAGTGCTGTCTGCCTAAGTTAACACCTTAAGCATCCCGCCTGGGGAGTACGGCCGCAAGGCTAAAACTCAAAGG
AATAGACGGGGACCCGCACAAGCGGTGGAGCATGTGGTTTAATTCGACGATAAGCGTGGAACCTCACCAAGGTT
TGACATATAGCTGCAGACCCCTGGAAACAGGGGAGCCTTCGAGGGTGCTATACAGATGCTGCATGGTTGTCGTC
AGCTCGTGTCGTGAGATGTACCGTTAAGTCGGGAAACGAGCGCAACCCCTACCCTATGTTTTACGTGTCATAGG
GAACTGTCTGCTTTAAGCAGGAGGAAGGTGGGGATGACGTCAAATCAGCATGGTTCTTACACCTTGGGCGACAC
ACGTGCTACAATGGGAAGTACAAAGGGACGCCAAATCGTAAGATGGAGCAAATCCCAAAAAACTTCCCTCAGTTC
GGATTGAGGGCTGCAACTCGCCCTCATGAAGCTGGAATCGCTAGTAAACGCAGATCAGCCATGCTGCGTTGAAT
ACGTTCTCGGGTCTTGTACTCACCGCCCGTCACACCAAGAGAGTCGGAGGCGCCCGAACTTGCTATTGCAGCAA
GGAAGGTGAAGCCGATGATAAGGGTGAAGTCGTAACAAGGCATCCGTAGCGGAAGCTGTGGATGGATCACCTC
CTTT
